# Brigatinib pharmacokinetics in patients with chronic hepatic impairment

**DOI:** 10.1007/s10637-023-01339-6

**Published:** 2023-04-13

**Authors:** Michael J. Hanley, David Kerstein, Meera Tugnait, Narayana Narasimhan, Thomas C. Marbury, Karthik Venkatakrishnan, Neeraj Gupta

**Affiliations:** 1 Takeda Development Center Americas, Inc., 95 Hayden Avenue, Lexington, MA 02421 USA; 2 Millennium Pharmaceuticals, Inc., a wholly owned subsidiary of Takeda Pharmaceutical Company Limited, Cambridge, MA USA; 3 Present Address: Theseus Pharmaceuticals, Cambridge, MA USA; 4 Present Address: Cerevel Therapeutics, Cambridge, MA USA; 5 Orlando Clinical Research Center, Orlando, FL USA; 6 Present Address: EMD Serono Research and Development Institute, Inc., Billerica, MA USA

**Keywords:** Brigatinib, Anaplastic lymphoma kinase, Tyrosine kinase inhibitor, Non-small cell lung cancer, Pharmacokinetics, Hepatic impairment

## Abstract

Brigatinib is an anaplastic lymphoma kinase (ALK) inhibitor approved for the treatment of *ALK*-positive non-small cell lung cancer. This open-label, parallel-group study investigated the effect of chronic hepatic impairment on the pharmacokinetics (PK) of brigatinib to inform dosing recommendations for these patients. Participants with hepatic impairment classified according to Child-Pugh categories of mild (A), moderate (B), or severe (C) and matched-healthy participants with normal hepatic function received a single oral dose of 90-mg brigatinib. Plasma samples were collected for the determination of brigatinib plasma protein binding and estimation of plasma PK parameters. Twenty-seven participants were enrolled (Child-Pugh A–C, n = 6 each; matched-healthy participants, n = 9). The mean fraction of free plasma brigatinib was comparable for the Child-Pugh A (11.1%), Child-Pugh B (10.8%), and healthy participant groups (8.5%); free brigatinib was higher in the Child-Pugh C group (23.1%). There were no clinically meaningful effects of mild or moderate hepatic impairment on unbound systemic exposures (area under the plasma concentration-time curve [AUC]) of brigatinib (geometric least-squares mean ratios [90% CI] of 89.32% [69.79%–﻿114.31%] and 99.55% [77.78%–127.41%], respectively). In the severe hepatic impairment group, brigatinib unbound AUC was approximately 37% higher (geometric least-squares mean ratio [90% CI] of 137.41% [107.37%–175.86%]) compared with healthy participants with normal hepatic function. Brigatinib was well tolerated in healthy participants and in participants with hepatic impairment. No dose adjustment is required for patients with mild or moderate hepatic impairment. The brigatinib dose should be reduced by approximately 40% for patients with severe hepatic impairment.

## Introduction

Activating rearrangements of the anaplastic lymphoma kinase gene (*ALK*) occur in 3–5% of non-small cell lung cancers (NSCLC) [1–3]. Brigatinib is a next-generation tyrosine kinase inhibitor (TKI) with selective activity against *ALK* and *ROS1* fusions. Brigatinib is approved for the treatment of adult patients with *ALK*-positive metastatic NSCLC based on the results of the phase 2 ALK in Lung Cancer Trial of BrigAtinib (ALTA) (NCT02094573) [4, 5] and the phase 3 ALTA in 1st Line study (ALTA-1L) (NCT02737501) [6]. In the final analysis in patients who were randomized to receive brigatinib 180 mg once daily (QD) with a 7-day lead-in at 90 mg QD in ALTA, with a median follow-up of 28.3 months, the independent review committee (IRC)-assessed confirmed objective response rate (ORR) was 56%, and the IRC-assessed median progression-free survival (PFS) was 16.7 months [4]. In the global phase 3 ALTA-1L trial (NCT02737501), the primary endpoint, PFS, favored brigatinib compared with crizotinib in patients with TKI-naive *ALK*+ NSCLC (hazard ratio, 0.48 [95% confidence interval (CI), 0.35–0.66]; *P* < 0.0001) [6]. The recommended dosing regimen for brigatinib is 90 mg QD for the first 7 days followed by an increase to 180 mg QD. This step-up dosing regimen reduced the frequency of adverse events while maximizing the potential for efficacy and has demonstrated a favorable benefit–risk profile in both the first-line setting and in patients with *ALK*+ NSCLC refractory to prior ALK TKI therapy [7–9].

Brigatinib exhibits linear pharmacokinetics (PK) with dose-proportional systemic exposures observed over the dose range of 60–240 mg QD. The mean plasma terminal elimination half-life of brigatinib is approximately 25 hours [10, 11]. A food-effect study conducted in healthy participants found no clinically meaningful effect of a high-fat, high-calorie meal on the total systemic exposure (AUC) of brigatinib versus administration under fasted conditions [12].

In vitro studies indicate that brigatinib is primarily metabolized by cytochrome P450 (CYP) 2C8 and CYP3A4 [13]. However, the results of a clinical drug-drug interaction study showed that CYP2C8 is not a meaningful determinant of brigatinib clearance in vivo as a strong CYP2C8 inhibitor had no clinically relevant effect on brigatinib systemic exposure [14]. In contrast, the strong CYP3A inhibitor itraconazole increased brigatinib area under the plasma concentration–time curve from time 0 to infinity (AUC_0−∞_) by 101%, while the strong CYP3A inducer rifampicin decreased brigatinib AUC_0−∞_ by 80%, thereby confirming the importance of CYP3A-mediated metabolism to brigatinib clearance [14]. Furthermore, in healthy participants who received a single oral dose of 180 mg [^14^C]-brigatinib, approximately 65% of the administered dose was recovered in the feces (41% as unchanged brigatinib) and 25% was recovered in the urine (86% as unchanged brigatinib) [15], indicating that hepatic metabolism is a primary contributor to total clearance. In a population PK analysis, age, race, sex, and body weight were found to have no clinically meaningful effect on brigatinib PK [15]. In addition, this population PK analysis demonstrated that aspartate aminotransferase, alanine aminotransferase, and total bilirubin did not meaningfully explain variability in brigatinib clearance, suggesting no brigatinib dose adjustment is required for these liver function–related covariates [15]. Notably, PK data from patients with moderate or severe hepatic impairment were not available for inclusion in this population PK analysis because these patients were excluded from trials conducted during clinical development. Given the role of CYP3A-mediated metabolism to brigatinib clearance and the limited PK data available for patients with chronic hepatic impairment, this present study was conducted to characterize the PK of brigatinib in patients with chronic hepatic impairment, classified according to the Child-Pugh criteria [16], and matched healthy participants with normal hepatic function to inform dosing recommendations for patients with mild, moderate, or severe hepatic impairment.

## Methods

### Study design

This open-label, parallel-group, inpatient, nonrandomized study was performed at 1 clinical study site, the Orlando Clinical Research Center (Orlando, FL, USA), and evaluated the PK of brigatinib after administration of a single oral 90-mg dose in participants with chronic hepatic impairment and in healthy participants with normal hepatic function. The study consisted of a screening period of up to 22 days and a single treatment and assessment period that included a 10-day (9-night) inpatient stay (Day −1 to Day 9). On the morning of Day 1, each participant received a single oral dose of 90 mg brigatinib under fasting conditions with approximately 240 mL of water. Fasting was required for approximately 10 hours before study drug dosing, for approximately 4 hours postdose, and for at least 8 hours before clinical laboratory tests. Meals were standardized for all participants. Water was permitted ad libitum except for the period between approximately 1 hour before and 1 hour after dosing. Participants remained in the clinic through 192 hours postdose for PK sample collection and were discharged from the clinical research site on Day 9.

### Participants

Prior to study initiation, approval was obtained from the institutional review board of the study site and all participants provided written informed consent. This study was conducted in accordance with current Good Clinical Practice, the Declaration of Helsinki, the International Council for Harmonisation guidelines, and all applicable regulatory requirements.

Eligible participants included men or women of nonchildbearing potential, aged 18–70 years, body mass index (BMI) of 18.0–39.0 kg/m^2^, and a minimum weight of 50.0 kg at screening. Participants who smoked were eligible provided they did not exceed 5 cigarettes per day during the inpatient stay. Participants with hepatic impairment were classified according to Child-Pugh categories of mild (Child-Pugh A; total score of 5–6), moderate (Child-Pugh B; total score of 7–9), or severe (Child-Pugh C; total score of 10–15) hepatic impairment [16] based on physical examination findings and laboratory results assessed at screening. Permitted hepatic impairment etiology included chronic alcoholism, chronic viral hepatitis B or C, nonalcoholic steatohepatitis, autoimmune hepatitis, Wilson disease, alpha-1 antitrypsin deficiency, glycogen storage diseases, or galactosemia. Healthy participants with normal hepatic function were matched with participants with chronic hepatic impairment by age (± 10 years), sex, BMI (± 15% at screening), and if possible, comparable smoking habits. One healthy participant was to be matched by these criteria for approximately every 2 participants with hepatic impairment in all 3 categories combined. Healthy participants with normal hepatic function were free from clinically significant abnormalities based on medical history, vital signs, physical examination, 12-lead electrocardiogram, and laboratory evaluations at screening, per investigator assessment.

Investigational or prescription drugs were not permitted within 30 days before study drug administration except for chronic stable medications taken by participants with hepatic impairment. Use of over-the-counter drugs or herbal supplements was prohibited, except for acetaminophen (≤ 2000 mg/day) and vitamins (≤ 100% of the recommended daily allowance), within 72 hours before study drug administration. Participants were required to abstain from alcohol, caffeine-containing products, grapefruit and grapefruit-containing products, pomegranate, pomelo, star fruit products, Seville oranges, quinine-containing food or drink, and poppy seeds from 72 hours before study drug administration until the end of the inpatient confinement period. Healthy participants with a surgical or medical condition (other than cholecystectomy) that could have potentially interfered with the absorption, distribution, metabolism, or excretion of brigatinib were excluded from study participation.

### Assessments

Blood samples were collected before brigatinib administration (predose) and at 0.5, 1, 1.5, 2, 2.5, 3, 4, 6, 8, 12, 24, 36, 48, 72, 96, 120, 144, 168, and 192 hours following brigatinib administration to measure brigatinib plasma concentrations. Samples to assess brigatinib plasma protein binding were collected at 2, 8, and 24 hours postdose.

### Bioanalytical methods

#### Plasma protein binding assay

Free fractions of brigatinib in plasma samples were determined as previously reported [17]. Briefly, free brigatinib was separated from protein-bound brigatinib by rapid equilibrium dialysis of plasma samples against warm (37° C) phosphate-buffered saline using the Thermo Scientific Single-Use Rapid Equilibrium Dialysis kit (Thermo Fisher Scientific, Waltham, MA) according to the manufacturer’s specifications. The semipermeable dialysis membrane had an 8000-dalton molecular weight cutoff. After incubation, samples were analyzed for brigatinib concentrations using liquid chromatography with tandem mass spectrometry as previously reported [14] using a dual-range assay with a lower limit of quantitation of 0.100 ng/mL and an upper limit of quantitation of 500 ng/mL.

#### Pharmacokinetics assay

Plasma samples were analyzed for brigatinib concentrations using a previously reported dual-range assay with a lower limit of quantitation of 0.100 ng/mL and an upper limit of quantitation of 2500 ng/mL [14].

### Safety

All enrolled participants who received brigatinib were included in the safety population. Adverse event (AE) monitoring occurred throughout the study. Physical examination was conducted at screening with additional symptom-directed physical examinations performed at the investigator’s discretion on study Days −1 and 9. Height, weight, and BMI were recorded at screening. Vital signs were recorded at screening; on Day −1; predose and at 0.5, 1, 2, 3, 4, 6, 8, 12, 24, 36, 48, 72, 96, 120, 144, and 168 hours postdose; and on Day 9. Electrocardiograms were conducted at screening, on Day −1, predose and at 3 and 48 hours postdose, and on Day 9. Clinical laboratory evaluations were performed at screening and on Days −1 and 9 and were reviewed by the investigator for clinical significance.

### Pharmacokinetic data analysis

The PK-evaluable population was defined as participants who received the single dose of brigatinib, had no major protocol deviations that affected the PK analyses, and had sufficient data to calculate PK parameters. Individual participant brigatinib plasma concentration-time profiles were analyzed via noncompartmental analysis using Phoenix WinNonLin version 6.4 (Certara, Inc., Princeton, NJ) to calculate plasma PK parameters. Plasma PK parameters calculated for brigatinib included maximum observed plasma concentration (C_max_), time to first maximum observed plasma concentration (T_max_), area under the plasma concentration-time curve from time 0 to the time of the last measurable concentration (AUC_0−last_), area under the plasma concentration-time curve from time 0 to infinity (AUC_0−∞_), terminal elimination half-life (t_1/2_), apparent oral clearance (CL/F), and apparent volume of distribution during the terminal disposition phase (V_z_/F). The fraction of unbound brigatinib in plasma was calculated using the following formula: % free (unbound) = [free brigatinib concentration measured in buffer after dialysis] / [total brigatinib concentration measured in plasma] × 100. Unbound brigatinib PK parameters were derived from the following formula (except for the unbound CL/F and unbound V_z_/F, which were calculated as total CL/F or total V_z_/F divided by the individual participant’s overall mean fraction unbound): unbound PK parameter = (PK parameter based on total concentrations) × (individual participant’s overall mean fraction unbound value).

### Statistical analysis

Descriptive statistics, including mean, standard deviation (SD), percent coefficient of variation (%CV), and median, minimum, and maximum values, were calculated for each time point for plasma brigatinib concentrations for the healthy participant and each Child-Pugh group. Arithmetic means, SD, %CV, median, minimum, and maximum values, and the number of observations were calculated for the PK parameters. Geometric mean and geometric %CV were derived for all parameters, except T_max_, which was reported as median (minimum–maximum). Statistical comparisons of unbound brigatinib PK parameters were made among the hepatic function groups (ie, healthy participants, Child-Pugh classes A, B, and C) using an analysis of variance. The geometric least-squares mean ratio (GMR) and 90% CIs were calculated for the comparison of C_max__,__u_, AUC_0 − last__,__u_, and AUC_0−∞,u_ between each Child-Pugh group and the healthy participants with normal hepatic function group. Safety outcomes were summarized using descriptive statistics.

## Results

### Demographics and baseline characteristics

Twenty-seven participants were enrolled in the study, including 6 participants in each of the 3 Child-Pugh hepatic impairment categories and 9 matched healthy participants with normal hepatic function. No participants were excluded from the PK-evaluable or safety analysis populations, and all participants completed the study. Demographics and baseline characteristics were generally similar among the hepatic function groups (Table 1).

**Table 1:** Demographics and baseline characteristics

**Characteristic**	**Normal** **Hepatic Function (n=9)**	**Mild** **(Child-Pugh A)** **Hepatic Impairment (n=6)**	**Moderate** **(Child-Pugh B)** **Hepatic Impairment (n=6)**	**Severe** **(Child-Pugh C)** **Hepatic Impairment (n=6)**
Age, median (range), years	57.0 (44–65)	54.5 (49–60)	58.0 (48–62)	59.0 (53–65)
Male, n (%)	7 (77.8)	5 (83.3)	6 (100)	3 (50.0)
Race, n (%)				
White	8 (88.9)	5 (83.3)	6 (100)	6 (100)
Black or African American	1 (11.1)	0	0	0
Asian	0	1 (16.7)	0	0
Ethnicity, n (%)				
Hispanic or Latino	5 (55.6)	3 (50.0)	2 (33.3)	1 (16.7)
Not Hispanic or Latino	4 (44.4)	3 (50.0)	4 (66.7)	5 (83.3)
BMI, mean (SD), kg/m^2^	28.7 (3.68)	29.0 (3.02)	30.7 (4.56)	30.3 (2.74)
Smoking history, n (%)				
Never smoked	4 (44.4)	0	3 (50.0)	2 (33.3)
Currently smoking	4 (44.4)	4 (66.7)	3 (50.0)	2 (33.3)
Formerly smoked	1 (11.1)	2 (33.3)	0	2 (33.3)

*BMI* body mass index, *SD* standard deviation

Participant age ranged from 44 to 65 years across all groups, with a median of 57.0 years in the healthy participant group, and a median of 54.5, 58.0, and 59.0 years in the mild, moderate, and severe hepatic impairment groups, respectively. Most participants were white (93%) and male (78%).

### Plasma protein binding of brigatinib

**Fig. 1:**
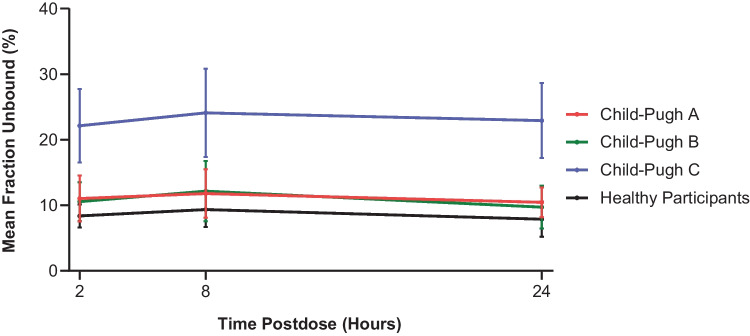
Mean (± standard deviation) plasma fraction unbound for brigatinib at 2, 8, and 24 hours postdose by hepatic function group

Within each hepatic function group, the mean fraction unbound for brigatinib was similar at 2, 8, and 24 hours postdose, indicating that protein binding was not concentration-dependent (Fig. 1).﻿ Consequently, plasma protein binding data were pooled across the 3 time points for each hepatic function group. The pooled mean ± SD fraction bound was comparable between the healthy participants (91.5% ± 2.3%), Child-Pugh A (88.9% ± 3.1%), and Child-Pugh B (89.2% ± 3.5%) groups, but was lower in the Child-Pugh C group (76.9% ± 5.7%). Accordingly, the mean fraction unbound value in the Child-Pugh C group (23.1%) was 2.7-fold higher than the unbound value observed in healthy participants (8.5%). Free plasma brigatinib in the Child-Pugh A (11.1%) and Child-Pugh B (10.8%) groups was not meaningfully different from that observed in healthy participants.

### Plasma pharmacokinetic parameters for brigatinib

The median (range) brigatinib T_max_ was similar across the healthy participant (2.00 h [1.00‒6.00 h]), Child-Pugh A (1.75 h [1.00‒3.00 h]), and Child-Pugh B (2.00 h [0.50‒3.00 h]) groups but was shorter in the Child-Pugh C group (0.50 h [0.50‒3.00 h]). The geometric mean brigatinib t_1/2_ was comparable across the hepatic function groups (47.6, 47.0, 48.7, and 51.0 hours for healthy participants, Child-Pugh A, Child-Pugh B, and Child-Pugh C, respectively).

**Fig. 2:**
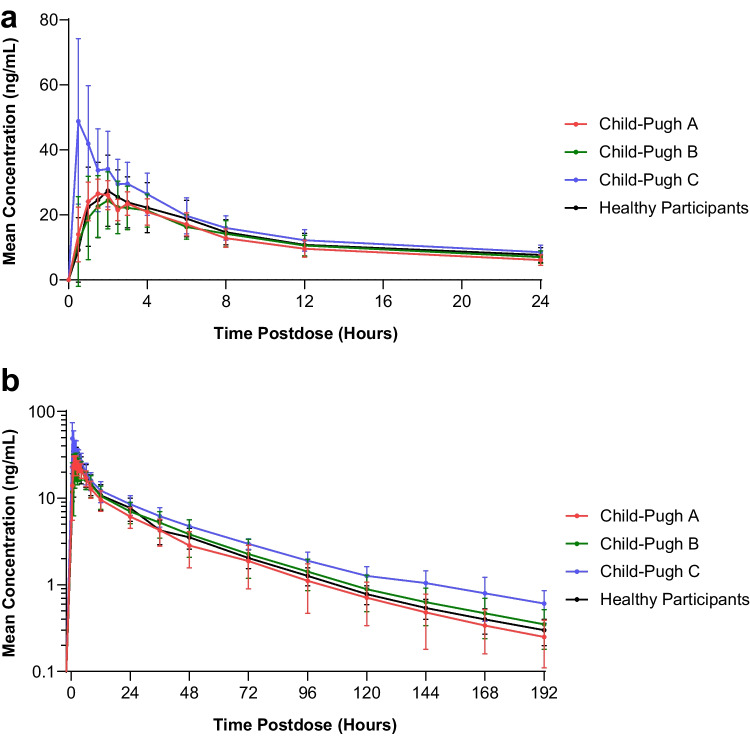
Mean (± ﻿standard deviation) unbound brigatinib plasma concentration-time profiles from **a** 0 to 24 hours postdose (linear scale) and **b** 0 to 192 hours postdose (log-linear scale)

The mean plasma concentration-time profiles for unbound brigatinib in each hepatic function group are shown in Fig. 2, and a summary of unbound PK parameters is provided in Table 2.﻿﻿ A statistical comparison of unbound brigatinib plasma PK parameters in participants with hepatic impairment versus healthy participants with normal hepatic function is presented in Table 3.

**Table 2:** Plasma PK parameters for brigatinib

**Parameter** ^**a**^	**Normal** **Hepatic Function (n=9)**	**Mild** **(Child-Pugh A)** **Hepatic Impairment (n=6)**	**Moderate** **(Child-Pugh B)** **Hepatic Impairment (n=6)**	**Severe** **(Child-Pugh C)** **Hepatic Impairment (n=6)**
T_max_ (h)	2.00 (1.00‒6.00)	1.75 (1.00‒3.00)	2.00 (0.50‒3.00)	0.50 (0.50‒3.00)
C_max__,__u_ (ng/mL)	30.3 (27.2)	28.7 (9.5)	27.8 (24.8)	50.0 (34.5)
AUC_0−last__,__u_ (h·ng/mL)	581 (25.0)	522 (24.6)	578 (38.3)	781 (20.7)
AUC_0−∞__,__u_ (h·ng/mL)	603 (25.0)	539 (24.9)	601 (39.8)	829 (20.0)
CL/F_u_ (L/h)	149 (25.0)	167 (24.9)	150 (39.8)	109 (20.0)
V_z_/F_u_ (L)	10,200 (31.0)	11,300 (45.8)	10,500 (29.5)	7,990 (33.2)
t_1/2_ (h)	47.6 (29.1)	47.0 (30.9)	48.7 (22.0)	51.0 (26.1)

*AUC*_*0−∞,u*_ unbound area under the plasma concentration-time curve from time 0 to infinity, *AUC*_*0−last,u*_ unbound area under the plasma concentration-time curve from time 0 to the time of the last measurable concentration, *CL/F*_*u*_ unbound apparent oral clearance, *C*_*max,u*_ unbound maximum observed plasma concentration, *PK* pharmacokinetic, *t*_*1/2*_ terminal elimination half-life, *T*_*max*_ time to first maximum observed plasma concentration, *V*_*z*_* / F*_*u﻿*_ unbound apparent volume of distribution during the terminal disposition phase

^a^Data are presented as geometric mean (geometric % coefficient of variation) with the exception of T_max_, which is presented as the median (range)

**Table 3:** Comparison of unbound brigatinib plasma PK parameters in the hepatic impairment groups versus the healthy participants with normal hepatic function group

**Parameter**	**Comparison** **(vs normal hepatic function)**	**Geometric LSM ratio, %** **(90% CI)**
C_max,u_ (ng/mL)	Mild (Child-Pugh A) hepatic impairment	94.75 (75.36–119.12)
	Moderate (Child-Pugh B) hepatic impairment	92.01 (73.18–115.68)
	Severe (Child-Pugh C) hepatic impairment	165.26 (131.44–207.78)
AUC_0−last,u_ (h·ng/mL)	Mild (Child-Pugh A) hepatic impairment	89.77 (70.37–114.52)
	Moderate (Child-Pugh B) hepatic impairment	99.41 (77.93–126.82)
	Severe (Child-Pugh C) hepatic impairment	134.38 (105.34–171.43)
AUC_0−∞,u_ (h·ng/mL)	Mild (Child-Pugh A) hepatic impairment	89.32 (69.79–114.31)
	Moderate (Child-Pugh B) hepatic impairment	99.55 (77.78–127.41)
	Severe (Child-Pugh C) hepatic impairment	137.41 (107.37–175.86)

*AUC*_*0−∞,u*_ unbound area under the plasma concentration-time curve from time 0 to infinity, *AUC*_*0−last,u*_ unbound area under the plasma concentration-time curve from time 0 to the time of the last measurable concentration, *CI* confidence interval, *C*_*max,u*_ unbound maximum observed plasma concentration, *LSM* least-squares mean, *PK* pharmacokinetic

Following oral administration of a single dose of brigatinib 90 mg, unbound brigatinib systemic exposures were comparable among the healthy participants with normal hepatic function, Child-Pugh A group, and Child-Pugh B group but were higher in the Child-Pugh C group. Specifically, the geometric mean brigatinib C_max,u_, AUC_0−last,u_, and AUC_0−∞,u_, were 65% (GMR [90% CI]: 165.26% [131.44–207.78%]), 34% (GMR [90% CI]: 134.38% [105.34–171.43%]), and 37% (GMR [90% CI]: 137.41% [107.37–175.86%]) higher, respectively, in the Child-Pugh C group compared with the healthy participants with normal hepatic function group.

### Safety

One or more treatment-emergent adverse events (TEAEs) were reported in 13 study participants, including 2 participants (2 events) in the Child-Pugh A group; 4 participants (13 events) in the Child-Pugh B group; 2 participants (2 events) in the Child-Pugh C group; and 5 participants (5 events) in the healthy participant group. Most TEAEs were mild in severity per investigator assessment. TEAEs observed in ≥ 2 participants were headache (4 participants), nausea, dyspnea, hypertension, and constipation (2 participants each). No clinically significant changes in laboratory values or electrocardiogram measurements were observed during the study. There were no serious AEs during the study, and no participants discontinued the study due to an AE.

Two participants in the Child-Pugh B group reported moderate TEAEs possibly related to the study drug. One participant experienced dyspnea and pyrexia, along with headache and vomiting. The participant was treated with supplemental oxygen for dyspnea; no medical intervention was received for the other TEAEs. The second participant experienced hypertension within 2 hours after brigatinib administration that lasted for approximately 2 days. The participant recovered without medical intervention. Both participants completed the study.

## Discussion

Collectively, the available data from the mass balance study and a clinical drug-drug interaction study indicate that CYP3A-mediated metabolism is the primary contributor to brigatinib clearance, with a minor contribution from renal elimination [11, 13, 14]. Thus, the present study was conducted to evaluate the effect of varying degrees of chronic hepatic impairment, defined using the Child-Pugh criteria, on the PK of brigatinib to inform dosing recommendations in these patient populations. Brigatinib exhibits linear and time-independent PK. Accordingly, this supported the utilization of a single-dose design and a 90-mg dose of brigatinib because the observed effects of chronic hepatic impairment on brigatinib AUC_0−∞,u_ in this study would be expected to be consistent with the effects of chronic hepatic impairment on brigatinib AUC at steady-state after QD administration of 180 mg brigatinib. Furthermore, the 90-mg brigatinib dose was anticipated to provide an adequate safety margin to account for increased systemic exposures in the setting of chronic hepatic impairment.

Brigatinib plasma protein binding was measured in this study and found to be similar across the 3 time points evaluated within each hepatic function group. This finding indicates that protein binding is not concentration-dependent, thereby corroborating the protein binding results from a previously reported renal impairment study [17]. Mild (Child-Pugh A) and moderate (Child-Pugh B) hepatic impairment had no clinically meaningful effect on the plasma protein binding of brigatinib. However, the mean fraction unbound for brigatinib in the severe (Child-Pugh C) hepatic impairment group was 2.7-fold higher than the mean value for healthy participants (23.1% vs 8.5%, respectively). After single-dose administration, brigatinib C_max,u_ and AUC_0−∞,u_ were comparable among the Child-Pugh A, Child-Pugh B, and healthy participant groups. Consequently, mild and moderate hepatic impairment have no clinically meaningful effect on brigatinib PK and no dosage adjustment is needed for these patients. In contrast, brigatinib C_max,u_ and AUC_0−∞,u_ in the Child-Pugh C group were approximately 65% and 37% higher, respectively, compared with the healthy participants with normal hepatic function group. Therefore, the results of this PK study indicate that the brigatinib QD dose should be reduced by approximately 40% (ie, from 180 mg to 120 mg, from 120 mg to 90 mg, or from 90 mg to 60 mg) for patients with severe hepatic impairment to provide brigatinib systemic exposures (AUC_0−∞,u_) comparable to those achieved in the setting of normal hepatic function.

The severity of hepatic dysfunction has been associated with the decreased expression of some CYP isoenzymes, including CYP3A [18–20]. In addition, liver disease is associated with alterations in hepatic blood flow, decreases in plasma proteins, and impaired biliary excretion [19, 21]. Thus, the increased brigatinib unbound exposure observed in the severe hepatic impairment group is likely the result of multiple pathophysiologic changes in these patients, including reduced CYP3A-mediated metabolism of brigatinib.

The effect of chronic hepatic impairment on brigatinib PK and corresponding dosing recommendations for these patients are generally consistent with the ALK inhibitors crizotinib, alectinib, and ceritinib [18, 22–26]. For crizotinib, concentrations increased in patients with moderate or severe hepatic impairment, resulting in the recommendation to reduce the crizotinib dose from 250 mg twice a day (BID) to 200 mg BID for patients with moderate hepatic impairment, and from 250 mg BID to 250 mg QD for patients with severe hepatic impairment [22, 23]. A clinical study of alectinib conducted in patients with chronic hepatic impairment and matched healthy participants with normal hepatic function showed that moderate hepatic impairment increased combined systemic exposures (AUC) of alectinib and its M4 metabolite by 36%, while severe hepatic impairment increased combined systemic exposures by 76% [18]. Based on these results, a reduced dose of 450 mg BID (from 600 mg BID) is recommended for patients with severe hepatic impairment [18, 24]. Systemic exposures of ceritinib were similar in study participants with mild and moderate hepatic impairment compared with healthy participants with normal hepatic function; however, unbound systemic exposure (AUC) was increased by 108% in participants with severe hepatic impairment [26]. The ceritinib dose for patients with severe hepatic impairment is therefore recommended to be reduced by approximately one third (eg, from 450 mg QD to 300 mg QD) [25, 26]. An evaluation of the effects of moderate or severe hepatic impairment on the PK of the ALK inhibitor lorlatinib has not been reported. As such, dosing recommendations have not yet been established for lorlatinib for patients with moderate or severe hepatic impairment [25, 27]. Finally, no dose modification is recommended for patients with mild hepatic impairment for any of the currently approved ALK inhibitors [13, 22–27].

## Conclusion

Mild and moderate hepatic impairment had no clinically meaningful effect on brigatinib PK, thereby indicating that no dose adjustment is required for these patients. Brigatinib unbound systemic exposure, as assessed by AUC_0−∞,u_, was 37% higher in the severe hepatic impairment group compared with the healthy participants with normal hepatic function group. This supports an approximate 40% reduction of the brigatinib QD dose (ie, from 180 mg to 120 mg, from 120 mg to 90 mg, or from 90 mg to 60 mg) for patients with severe hepatic impairment, as reflected in the brigatinib prescribing information [13]. Brigatinib administered as a single 90-mg dose was well tolerated in healthy participants and in participants with mild, moderate, or severe hepatic impairment.

## Data Availability

The data sets generated and/or analyzed during the current study are available from the corresponding author on reasonable request to researchers who provide a methodologically sound proposal. The data will be provided after their de-identification, in compliance with applicable privacy laws, data protection, and requirements for consent and anonymization.
